# Effectiveness of App-Based Cognitive Screening for Dementia by Lay Health Workers in Low Resource Settings. A Validation and Feasibility Study in Rural Tanzania

**DOI:** 10.1177/0891988720957105

**Published:** 2020-09-23

**Authors:** Stella-Maria Paddick, Marcella Yoseph, William K. Gray, Damas Andrea, Robyn Barber, Aofie Colgan, Catherine Dotchin, Sarah Urasa, John Kissima, Irene Haule, Aloyce Kisoli, Jane Rogathi, Ssenku Safic, Declare Mushi, Louise Robinson, Richard W. Walker

**Affiliations:** 1 Newcastle University, Newcastle upon Tyne, United Kingdom; 2 108095 Kilimanjaro Christian Medical University College, Moshi, Tanzania; 3 Northumbria Healthcare NHS Foundation Trust, North Tyneside General Hospital, North Shields, United Kingdom; 4 Mirembe National Hospital, Dodoma, Tanzania; 5 Hai District Hospital, Boman’gombe, Kilimanjaro, Tanzania; 6 Mount Meru Hospital, Arusha, Tanzania

**Keywords:** cognition, dementia, Tanzania, Sub-Saharan Africa, low- and middle-income countries

## Abstract

**Background:**

The majority of people with dementia live in low-and middle-income countries (LMICs). In sub-Saharan Africa (SSA) human-resource shortages in mental health and geriatric medicine are well recognized. Use of technological solutions may improve access to diagnosis. We aimed to assess the diagnostic accuracy of a brief dementia screening mobile application (app) for non-specialist workers in rural Tanzania against blinded gold-standard diagnosis of DSM-5 dementia. The app includes 2 previously-validated culturally appropriate low-literacy screening tools for cognitive (IDEA cognitive screen) and functional impairment (abbreviated IDEA-IADL questionnaire).

**Methods:**

This was a 2-stage community-based door-to-door study. In Stage1, rural primary health workers approached all individuals aged ≥60 years for app-based dementia screening in 12 villages in Hai district, Kilimanjaro Tanzania.

In Stage 2, a stratified sub-sample were clinically-assessed for dementia blind to app screening score. Assessment included clinical history, neurological and bedside cognitive assessment and collateral history.

**Results:**

3011 (of 3122 eligible) older people consented to screening. Of these, 610 were evaluated in Stage 2. For the IDEA cognitive screen, the area under the receiver operating characteristic (AUROC) curve was 0.79 (95% CI 0.74-0.83) for DSM-5 dementia diagnosis (sensitivity 84.8%, specificity 58.4%). For those 358 (44%) completing the full app, AUROC was 0.78 for combined cognitive and informant-reported functional assessment.

**Conclusions:**

The pilot dementia screening app had good sensitivity but lacked specificity for dementia when administered by non-specialist rural community workers. This technological approach may be a promising way forward in low-resource settings, specialist onward referral may be prioritized.

## Introduction

Dementia disproportionately affects low and middle income countries (LMICs)^1^ and demographic transition and population ageing are predicted to result in a rapid and ongoing increase in those affected. In sub-Saharan Africa (SSA), as in many other LMIC settings, human-resource shortages in mental health and geriatric medicine are well recognized, particularly in rural areas.^2-5^ This can present challenges in access to healthcare leading to a substantial diagnostic and treatment gap for people with dementia and other non-communicable diseases (NCDs), as previously demonstrated by our team in Tanzania.^6-8^ Identification of dementia and receiving a diagnosis is felt to be beneficial in terms of allowing family members and the wider community to understand the symptoms and to allow future care planning and referral for dementia-specific intervention.^9,10^ To help increase rates of diagnosis and access to support, task-shifting, where non-specialist healthcare workers are mobilized to address this “mental health gap,” is the recommended approach in LMIC settings.^5,11,12^ Despite this, there is a current lack of validated dementia screening and case finding tools appropriate for LMIC settings.^13,14^

Use of technological solutions may improve access to diagnosis, particularly in the context of a task-shifting approach. Mobile health (m-health) and similar initiatives have been investigated in chronic disease management in similar low-resource settings^15^ and form part of current WHO recommendations for non-communicable diseases in low-resource settings.^16^ Potential advantages include streamlined data collection to inform policy makers, standardisation of advice and access to telemedicine for remote communities, but current evidence in SSA remains limited.^15^

Our team have previously-validated culturally appropriate low-literacy paper and pencil dementia screening tools for use in SSA, including the IDEA cognitive screen^17-19^ and IDEA-instrumental activities of daily living (IDEA-IADL) questionnaire for identification of functional impairment.^20,21^ We aimed to assess the diagnostic accuracy of a pilot brief dementia screening mobile application (app) based on these previously-validated tools. We also aimed to evaluate the feasibility and utility of community dementia screening by rural primary health workers in this setting by comparing door-to-door screening of all older people with a previous study based on recruitment at “screening days” held in the same rural setting to identify whether cases might be missed.

This study formed part of the NIHR Dementia Prevention and Care (DePEC) study of dementia risk reduction, screening and interventions in LMIC settings.

## Methods

### Study Setting

The study was conducted in the Hai demographic surveillance site (DSS), located in the Kilimanjaro region of Tanzania. The prevalence of dementia was estimated at 6.4% (age-adjusted) in those aged ≥70 in 2010.^22^ Within Kilimanjaro, there is a qualified psychiatrist (appointed 2019) at the local tertiary referral hospital (Kilimanjaro Christian Medical Centre) out of a total of ∼50 registered psychiatrists country-wide. Specialist old age psychiatry training is not currently available in Tanzania.

Hai demographic surveillance site (DSS) is a rural, well-demarcated area with a highly organized village structure facilitating epidemiological research and follow-up. There is substantial local experience in non-communicable disease research^23^ and a number of previous epidemiological and interventional studies of dementia and related conditions have taken place since 2010.^17,24-27^ The majority of the population are subsistence farmers, and a minority engage in commercial agriculture. Levels of illiteracy are high in older adults.^22^ For this study, 12 villages within Hai were randomly selected, stratified by altitude (highland, mid-zone, lowland). This stratification was intended to ensure representativeness by socioeconomic status, primarily driven by greater crop yields seen in highland areas.

### Ethics and Consent

A favorable ethical opinion was received from the Tanzanian National Institute of Medical Research and the Kilimanjaro Christian Medical University College Research Ethics Committee. For each participant, verbal information was provided about the aims of the study and the implications of taking part and a consent form was read aloud. Participants were also given the opportunity to ask questions. Consent was then obtained by signature or thumbprint, depending on literacy status. Where the participant was felt to lack capacity to give consent based on responses to study information, assent was sought from a close relative. Onward referral for identified clinical need during data collection was a key element of ethical consideration and appropriate procedures were agreed with the Hai District Medical Office.

### Study Design

**Figure 1.**
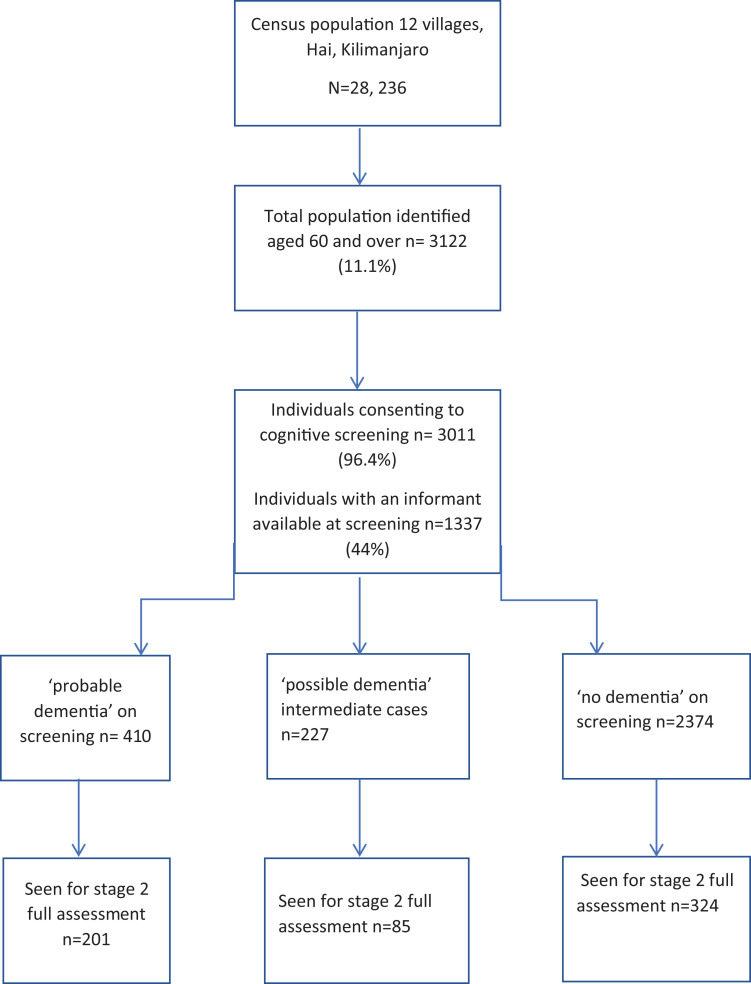
Study flowchart.

This was a 2-stage, blinded validation study. In stage 1, all consenting individuals aged ≥60 years were screened for cognitive impairment using the IDEA cognitive screen and abbreviated IDEA-IADL questionnaire combined and administered via the prototype app. The age cut-off was selected to allow evaluation in a broad selection of the population, and to reflect the standard public service retirement age in Tanzania.^28^ In stage 2, a stratified sample was then assessed against DSM-5 dementia criteria by consensus panel review of detailed clinical notes by psychiatrists and geriatricians with an interest in dementia, blinded to cognitive screening results. In stage 2, we aimed to assess all screen-positive participants (screening-defined “probable dementia”), alongside 50% of screen-borderline participants (screening-defined “possible dementia”) and 10% of screen-negative individuals (screening-defined “no dementia”) selected by random number generator. Individuals assessed at each stage are detailed in the study flowchart (see Figure 1). Stage 1 screening data were collected between 9th March and 28th July 2018 and stage 2 assessment data between 13th March 2018 and 26th April 2019

### Dementia Screening—Stage 1

Within the 12 selected villages, dementia screening was accompanied by a door-to-door census to ensure that all individuals aged ≥60 years were identified. Subsequent follow-up visits were made to identify those potentially aged ≥60 years who were not at home at the initial census visit.

Dementia screening was conducted by census enumerators; individuals holding core positions within the Hai DSS structure and resident within each village. Most enumerators were highly experienced in previous epidemiological data collection, though some were recently-appointed due to retirements and changes in village boundaries.

Prior to commencing data collection, enumerators were trained in administration of the screening measures and use of the app-based screening tools at a 2-day workshop. Training included information about dementia and the rationale for the study, alongside practical role-play exercises for screening and consent procedures with volunteer elders from the local community. Around half of the enumerators had been involved in recruitment within previous dementia studies and were familiar with the concept of dementia.

Determination of age is recognized to be challenging in rural SSA elders who may lack formal identification documents. Age was calculated from year of birth and triangulated using a table of historical events alongside ages at marriage and of first child (see supplementary online materials for table used). This method is well-validated in SSA^29,30^ and was also used in a previous (2010) dementia prevalence study in Hai.^22^

Within villages, screening was overseen and organized by senior members of the research team, experienced in previous dementia studies and who were both from the local area (AK, JK). Newly appointedenumerators (n = 2) received one to one support for the first few days of data collection. Data collection forms and instructions were translated into Kiswahili (the lingua franca) and any areas requiring clarification refined at the initial study workshop. All enumerators spoke both Swahili and local tribal languages (Chagga and Maasai, dependent on village) and translated verbally to participants not fluent in Kiswahili.

### Screening for Dementia—Prototype App

The prototype app used for screening was developed using data collection forms developed in Open Data Kit (ODK) software on hand-held Android tablets. The app was used to administer the IDEA cognitive screen^18^ and the abbreviated IDEA-IADL questionnaire,^20,21^ both previously validated in Tanzania individually and as combined paper-and pencil tests. Questions on self-rated subjective cognitive impairment (SCI) were also included. Specifically, individuals were asked “Has your memory become worse over the last year?” and, if answered affirmatively, “Do your memory difficulties interfere with your everyday life?” Questions on demographic background (age, occupational and educational background and household composition) were also included. Enumerators were able to specify difficulties interfering with screening (e.g. significant visual or auditory impairment) as free text answers.

Data were uploaded on a weekly basis to a secure, encrypted server, held by Kilimanjaro Clinical Research Institute, Moshi, Tanzania and quality-checked weekly by the study statistician (WKG) and JR. See **online supplementary materials** for the ODK variables used and questions included within the prototype App.

### Screening of Cognitive and Functional Impairment

The IDEA cognitive screen^18,19,31^ has been validated in a number of SSA settings, appears not to be educationally biased in low literacy settings and has been utilized as an outcome measure in SSA research studies. It incorporates items assessing abstraction, orientation, long term memory and categorical verbal fluency alongside the Consortium to Establish a Registry for Alzheimer’s Disease (CERAD) 10-word recall test,^32^ widely validated in LMIC settings and a literacy-independent matchstick visuo-construction task, initially developed and validated in Nigeria^33^). For the whole scale, the maximum possible score is 15 and the minimum 0, with a higher score indicating better cognitive function. Previous validation work in Tanzania has established the following categorizations: 0-7 (low performance), 7-9 (intermediate/borderline performance) and 10-15 (good performance)

A brief, previously validated version of the IDEA-IADL questionnaire was also administered.^20,21^ Previous validation work indicates good diagnostic accuracy for dementia used alone, and alongside cognitive screening.^20,21^ The abbreviated version of this culturally-appropriate, locally-developed questionnaire includes 3 informant-answered questions on functional ability Each item is scored as 0 (cannot do), 1 (can do with assistance) and 2 (can do easily), giving a total possible score of 6 in those with intact IADLs. The possible score on the combined cognitive and IADL screen therefore ranges from 0 to 21.

### Stratification for Full Assessment in Stage 2

In those with an informant, screening performance was stratified according to combined performance on the IDEA cognitive screen and the abbreviated IDEA-IADL questionnaire based on previous paper and pencil validation (score 0-10: categorized “probable dementia,” score 11-13: categorized “possible dementia,” score 14-21: categorized “no dementia”).^20^ In those without an informant, stratification was based only on performance on the IDEA cognitive screen (score 0-7: “probable dementia,” score 8-9: “possible dementia,” score 10-15: “no dementia”).

### Stage 2 Blinded Clinical Assessment for Dementia

Structured clinical assessments for dementia in stage 2 were completed by a separate team blinded to outcome of initial app-based screening.

Clinical neurocognitive assessments were completed and documented by MY (a Tanzanian post-internship doctor, with previous experience of dementia diagnosis in a research context)) and UK medical students, RB and AC, locally supervised by DA (a Tanzanian psychiatrist). Stage 2 assessment was based on the DSM-5 criteria. A detailed case history was prepared based on face to face clinical assessment. This included a repeat paper-and-pencil IDEA cognitive screen (to identify cases of recent marked cognitive change), alongside a structured protocol previously used in other Tanzanian studies.^34^ This included structured informant history, bedside cognitive examination, confusion assessment method (CAM) for delirium, neurological examination, 15-item Geriatric Depression Scale and structured mental state examination. Clinicians were able to ask and document additional clinical questions to exclude non-dementia causes of cognitive impairment (e.g. psychiatric disorders, learning disability) and produced a detailed written summary indicating provisional diagnosis and reasoning. A selection of individuals were clinically reviewed in person by DA, to confirm (or refute) provisional diagnoses and provide clinical supervision to those completing assessments. Complete case summaries were subsequently independently reviewed (S-MP, RW, CD) to confirm DSM-5 dementia criteria.

### Collection of Feasibility and Acceptability Data

Feasibility data were collected by RB and AO from the first 84 older individuals seen for stage 2 clinical assessments and from the 12 enumerators completing the screening process. Responses were collected on self-rated paper-and pencil questionnaires forward and back-translated from English to Kiswahili utilizing Likert scales and free text comments. In the case of older individuals unable to read or write, a literate relative was asked to read out the questions and document responses. Free text responses were subsequently translated to English by a core member of the team not involved in this aspect of data collection (MY). Questionnaires were completed anonymously.

### Comparison to 2014 “Dementia Screening Day” Initiatives

Demographic characteristics of participants were compared to those of a community-based sample aged ≥65 years (n = 466) who presented for cognitive assessment in 2014 as part of a widely advertised dementia screening program within the same geographical area of the Hai demographic surveillance site.^17,21^ This formed part of a dementia awareness program in collaboration with local community and religious leaders and the Hai District Medical Office as part of the Identification and Interventions for Elderly Africans (IDEA) study. This cohort self-presented to designated screening centers or were assessed at home where mobility was poor.

### Statistical Analysis

Statistical analyses were supported by IBM SPSS for Windows version 21 (IBM Corp, Armonk, NY, USA) and SAS (SAS Institute Inc, Cary, NC, USA). Descriptive statistical analysis used standard summary measures depending on the nature of the data (parametric and non-parametric). The area under the receiver operating characteristic (AUROC) curve statistic was used to assess diagnostic accuracy, alongside sensitivity, specificity, positive predictive value and negative predictive value.

## Results

The census population of the 12 villages was 28,236, of whom 3122 (11.1%) were aged ≥60 years and 3011 (96.4%) consented to participate (see Figure 1). Of those screened, 1,337 (44.4%) had an informant able to complete the IDEA-IADL questionnaire at initial screening. A total of 610 individuals were seen for stage 2 clinical assessment (201/410 screen-probable dementia, 85/227 intermediate performance and 324/2374 screen-negative individuals) seen in order of availability. Of these, 358 (58.7%) had an informant present at the time of stage 2 assessment.

### Diagnostic Accuracy

Summary statistics for the diagnostic accuracy of the combined IDEA cognitive screen and abbreviated IDEA-IADL questionnaire and for the 2 screens and their individual items in isolation are presented in **Table 1**. The combined IDEA cognitive screen and abbreviated IDEA-IADL questionnaire had an AUROC of 0.779, a sensitivity of 87.8%, specificity of 51.4%, positive likelihood ratio of 1.81 and negative likelihood ratio of 0.24. The AUROC for the IDEA cognitive screen used on its own was slightly higher at 0.789%, with a sensitivity of 84.8% and a specificity of 58.4%, positive likelihood ratio of 2.04 and negative likelihood ratio of 0.26. The most accurate individual items were delayed recall and categorical verbal fluency. AUROC values were lower in females, those without formal education, and older adults (see **Table 2**).

**Table 1.** AUROC Curve for Total Scores and Individual Items on IDEA Cognitive Screen and Abbreviated IDEA IADL Questionnaire.

	Maximum possible score	AUROC (95% CI)	Cut-off	Sensitivity	Specificity	Positive likelihood ratio	Negative likelihood ratio
IDEA cognitive screen	15	78.9 (74.6 to 83.1)	≤ 7	84.8%	58.4%	2.04	0.26
Abbreviated IDEA-IADL	6	71.9 (66.1 to 77.5)		–	–	–	–
Combined IDEA and abbreviated IDEA-IADL	21	77.9 (72.5 to 83.3)	≤ 10	87.8%	51.4%	1.81	0.24
Individual screening items							
Immediate word recall: total	30	72.6 (67.9 to 77.4)					
Delayed word recall	10	72.1 (68.0 to 76.2)					
Animal naming	–	72.2 (67.4 to 77.0)					
Matchstick visuospatial construction	3	67.0 (62.2 to 71.8)					
Presides over feasts and ceremonies	2	66.1 (61.4 to 70.7)					
Gives advice	2	68.2 (62.4 to 74.1)					
Does small works around the house	2	68.5 (62.7 to 74.2)					

**Table 2.** Median IDEA Cognitive Screen Values and AUROC for the IDEA Cognitive Screen by Age, Gender and Education.

	Screened for dementia	Median IDEA cognitive screen score (IQR)	Assessed for presence of dementia (n = 610)	Dementia diagnosis (n = 105)	AUROC (95% CI)
Age band					
60-64 years	775	13 (12 to 14)	87	1	Not calculated, only 1 positive case
65-69 years	622	13 (12 to 14)	77	3	0.811 (0.675 to 0.947)
70-74 years	513	12 (11 to 14)	100	4	0.799 (0.671 to 0.928)
75-79 years	437	12 (10 to 13)	93	20	0.779 (0.667 to 0.891)
80-84 years	299	11 (7 to 13)	104	31	0.676 (0.559 to 0.793)
85 years and over	365	9 (5 to 12)	149	46	0.735 (0.653 to 0.816)
Sex					
Female	1726	12 (10 to 14)	401	75	0.783 (0.728 to 0.837)
Male	1285	13 (11 to 14)	209	30	0.800 (0.736 to 0.863)
Education*					
No formal education	778	10 (7 to 14)	273	68	0.749 (0.687 to 0.811)
Some formal education	2225	13 (11 to 14)	332	35	0.799 (0.730 to 0.868)

* Eight people who were screened and 5 people who were assessed for dementia had no education level recorded and were excluded from this analysis

### Diagnostic Accuracy in Individuals With Subjective Cognitive Impairment

AUROC curves for diagnostic accuracy of the DePEC App in those with self-rated subjective cognitive impairment (therefore potentially more likely to self-present at health screening events) were similar to those for the entire sample assessed. SCI was highly prevalent. A total of 1888 (62.7%) of individuals answered affirmatively to “has your memory worsened over the past year,” and of these 1040 (34.5%) individuals endorsed “memory causes difficulties with activities of daily living.” Of those, AUROC for dementia using the App-based screening was 75.0% for those endorsing the first and second SCI questions respectively.

### Feasibility and Acceptability

Feasibility questionnaires were completed by 68/84 individuals assessed in stage 2 (81%) and all 12 study enumerators. Most census enumerators (11/12) and older participants (63/68) preferred the app system to paper assessments. All screeners said the app would be useful for future work, though 25% said that regular charging of the tablet devices was challenging and 41.5% admitted to some app questions being confusing. The responses of enumerators are summarized in **Table 3**. There were no unsuccessfully completed screening App questionnaires uploaded. The median time for complete data collection (census and cognitive screening) was19.2 minutes (inter-quartile range 13-27).

**Table 3.** Responses of Enumerators (Rural Community Health Workers) to Feasibility and Acceptability Questions Regarding App-Based Dementia Screening.

	Strongly Agree	Agree	Neutral	Disagree	Strongly Disagree
The tablet was simple to use	3 (25%)	5 (41.7%	4 (33.3%)	0	0
The app was simple to navigate	2 (16.7%	4 (33.3%	6 (50%)	0	0
The questions assessing dementia were confusing	0	2 (16.7%)	4 (33.3%)	5 (41.7%)	1
The instructions given for the app were suitable and easy to understand	3 (25%)	6 (50%)	3 (25%)	0	0
It was difficult to keep the tablet charged	0	3 (25%)	3 (25%)	4 (33.3%)	2 (16.7%)

### Comparison of Door-to-Door Dementia Screening and Population Attending Advertised Screening Events

Basic demographic data of those screened, are compared to previous results obtained through widely-advertised “screening days” in the same setting in 2014 in Table 4. The oldest-old (aged ≥85 years), those without formal education, and those with cognitive impairment (IDEA cognitive screen score ≤7) appear less likely to attend “screening days” even when given the option to be assessed at home. This group did consent to house to house screening by locally-resident lay workers as part of this current study.

**Table 4.** Comparison of 2014 Community Dementia Screening Days (IDEA study) and 2018 Screened Census Population Selected From Hai of Those Aged ≥ 65 Years (DePEC Study).

	IDEA Dementia screening days 2010 (n = 466)	DePEC study, census screening 2018 (n = 2236)
Age band		
65-69 years	145 (31.1%)	622 (27.8%)
70-74 years	108 (23.2%)	513 (22.9%)
75-79 years	90 (19.3%)	437 (19.5%)
80-84 years	77 (16.5%)	299 (13.4%)
85 years and over	46 (9.9%)	365 (16.3%)
Sex		
Female	261 (56.0%)	1264 (56.5%)
Male	205 (44.0%)	972 (43.5%)
Education		
No formal education	87 (18.7%)	689 (30.8%)
Some formal education	371 (79.6%)	1547 (69.2%)
IDEA cognitive screen performance		
Scored ≤ 7	41 (8.8%)	356 (16.3%)
Scored 8-9	59 (12.7%)	85 (3.8%)
Scored 10-15	366 (78.5%)	1795 (78.9%)

## Discussion

We sought to evaluate alternative screening and case-finding methods for dementia in a rural SSA setting. Comparing dementia screening using this tablet-based door to door method and the previous pen-and-paper screening tool validation studies in those presenting to widely advertised screening days, it appears that older individuals, those with lower education and lower cognitive performance (within the “probable dementia” category) were less likely to attend screening and could therefore be missed by targeted screening. Our finding that diagnostic accuracy of the app-based screening tools appeared not to be higher in those with subjective cognitive impairment, supports this view.

Performance of the combined cognitive and functional assessment in terms of diagnostic accuracy for dementia was lower than when previously evaluated as full-length paper-and-pencil tests. One reason for this may be the differing, and younger, population surveyed (60 and over, compared to 65 and over in original validation studies). In younger populations with low dementia prevalence, other non-dementia causes of cognitive impairment and therefore false positives on screening become more likely. Another reason may be that screening at previous “dementia screening days” was conducted or supervised by qualified health workers (nurses, psychologists or occupational therapists) and that task-shifting to lay dementia screeners may not be able to achieve the same level of accuracy.

Previous validation of the abbreviated combined paper-and-pencil IDEA-IADL screen similarly suggested that performance may have been affected by previous training and experience of lay screeners.^20^ Task-shifting of mental illness and NCD identification and management is widely recommended due to well-evidenced human resource shortages.^5^ The evidence for use of this strategy in diagnosis of mental health conditions in this setting with rural/lay health workers is currently limited, although promising results have been shown for chronic disease management.^11^ Our results outline both the feasibility, and the limitations, of this approach and the need for supervision and diagnostic confirmation by more specialist clinicians. Nevertheless, a recent community based validation of another commonly-used multi-domain cognitive screening tool, the Kiswahili MOCA (K-MOCA) for dementia in rural Tanzania, confirms the lack of educational bias in IDEA screen and difficulty with using tools developed elsewhere in SSA.^35^

In low-resource settings, a decision has to be made whether to prioritize sensitivity (more likelihood of identifying cases, and early dementia, but risk of giving a false dementia diagnosis) or specificity (likely to identify dementia later, but with more certainty). In this study, sensitivity of this method remains high, (with lower specificity) indicating that onward confirmatory assessment is recommended.

This study indicates that if this strategy is to be used with lay health workers then a focus on case-finding in those more likely to have dementia (for example the oldest-old, those with lower education and those attending healthcare settings) may be more useful. A method of onward referral to clinicians with greater levels of training, whether face to face or using telemedicine type approaches is needed.

Four people were subsequently found to have dementia who had a normal cognitive screening score (IDEA >9/15). Detailed case summaries were supportive of dementia and repeat cognitive screening at the time of assessment was consistent with dementia. It seems likely that the delay between initial screening and confirmatory diagnosis may have resulted in some individuals having experienced substantial cognitive decline which was not evident at screening.

Although early diagnosis is valued in HICs, in LMIC settings this may vary depending on the availability of interventions. Acetylcholinesterase inhibitors remain difficult to access, prohibitively expensive for the majority of people, and do not currently appear in the Tanzanian national treatment guidelines or essential medicines list. Lack of routine brain imaging in this setting, or access to a dementia sub-type diagnosis, may also limit their usefulness. Cognitive stimulation therapy (CST) has been evaluated in Tanzania and Nigeria, appears feasible and beneficial and is becoming available in Tanzania.^25,26^ Carer interventions are also being evaluated and are likely to be beneficial, given the current stigma and lack of awareness of dementia observed among people with dementia and their carers in Tanzania.^36^ We would argue that an early diagnosis is also to be recommended in SSA to enable access to these interventions, and access to support for both people with dementia and carers as in HICs.

This was generally the first time enumerators had used a tablet device and, although there were some difficulties maintaining charge in the device, our feasibility data suggest that this was an acceptable and feasible strategy for data collection to a centralized server for both enumerators and participants. The high proportion of individuals consenting to screening from those residing in the included villages, including those less likely to attend screening days, supports use of community screening by locally-resident workers in patients’ homes. This would allow policy makers to accurately target resources and interventions to areas of need in future. Nevertheless, informal discussion and evaluation of this program by our team suggests that more extensive training, peer support and harmonization measures would be considered when utilizing this dementia screening method in future.

### Limitations

Unexpectedly severe weather conditions seriously impeded transport within Kilimanjaro from March-June 2018. This, and logistical difficulties in recruitment of suitably qualified clinicians for confirmatory dementia diagnosis, caused significant delays between stage 1 screening and subsequent confirmatory assessment. We mitigated this by repeating the IDEA screen as a paper-and -pencil test at the time of confirmatory assessment in order to identify individuals with markedly different cognition to that recorded in baseline screening. Due to the delay, a number of individuals were lost to follow-up; a number which was relatively low compared to similar follow-up studies, but higher than that normally achieved within the Hai DSS. It would have also been useful to measure inter-rater reliability between screeners in this setting. This was not feasible due to the adverse weather conditions but would have been useful in identifying variability between screeners.

Clinical assessments for dementia were also completed by different individuals with different levels of experience. We attempted to mitigate this by arranging training and supervision via local specialists based at Mirembe national psychiatric hospital in Tanzania, ensuring all clinicians adhered to a highly structured clinical examination protocol and standardized documentation and consensus panel review. There may have nevertheless been some subtle issues that were missed. In particular, we note the relatively high proportion of impairment in delayed recall on the CERAD 10-word list in those without dementia (Supplementary Table 1). It is likely that a proportion of these individuals met criteria for mild cognitive impairment (MCI), but a confident estimate of MCI prevalence was not possible given our study design. Similarly, though we screened for depression using the GDS, and subsequently assessed screen-positive individuals for DSM-5 major depression is it possible some cases were similarly missed, given our study focus on dementia.

## Conclusion

Dementia screening, via an app based technology, of all older people for diagnosing potential dementia by lay healthcare workers is feasible to replace pen-and-paper tools in rural SSA. Screening within rural communities has the advantage of including those at greatest risk (the oldest-old, those with likely cognitive impairment and lower educational level), a population who appear less likely to self-present for screening. It seems that diagnostic accuracy of any screening measure may remain limited in a task-shifting context. The focus of screening should be on maximizing sensitivity and routes of onward referral in those groups likely to be at greatest risk, or settings of highest dementia prevalence. These are likely to be the oldest old, those without formal education or hospital-based populations.

## Supplemental Material

Supplemental Material, App_revision_supplementary_tables - Effectiveness of App-Based Cognitive Screening for Dementia by Lay Health Workers in Low Resource Settings. A Validation and Feasibility Study in Rural Tanzania Click here for additional data file. Supplemental Material, App_revision_supplementary_tables for Effectiveness of App-Based Cognitive Screening for Dementia by Lay Health Workers in Low Resource Settings. A Validation and Feasibility Study in Rural Tanzania by Stella-Maria Paddick, Marcella Yoseph, William K. Gray, Damas Andrea, Robyn Barber, Aofie Colgan, Catherine Dotchin, Sarah Urasa, John Kissima, Irene Haule, Aloyce Kisoli, Jane Rogathi, Ssenku Safic, Declare Mushi, Louise Robinson and Richard W. Walker in Journal of Geriatric Psychiatry and Neurology

Supplemental Material, ODK_word_doc_screening_paper - Effectiveness of App-Based Cognitive Screening for Dementia by Lay Health Workers in Low Resource Settings. A Validation and Feasibility Study in Rural Tanzania Click here for additional data file. Supplemental Material, ODK_word_doc_screening_paper for Effectiveness of App-Based Cognitive Screening for Dementia by Lay Health Workers in Low Resource Settings. A Validation and Feasibility Study in Rural Tanzania by Stella-Maria Paddick, Marcella Yoseph, William K. Gray, Damas Andrea, Robyn Barber, Aofie Colgan, Catherine Dotchin, Sarah Urasa, John Kissima, Irene Haule, Aloyce Kisoli, Jane Rogathi, Ssenku Safic, Declare Mushi, Louise Robinson and Richard W. Walker in Journal of Geriatric Psychiatry and Neurology
