# Supplementary material for: Effectiveness of App-Based Cognitive Screening for Dementia by Lay Health Workers in Low Resource Settings. A Validation and Feasibility Study in Rural Tanzania
Source: J Geriatr Psychiatry Neurol. 2020 Sep 23;34(6):613–21. doi: 10.1177/0891988720957105 (PMC8600584; doi:10.1177/0891988720957105)
Supplement: Supplemental Material, App_revision_supplementary_tables - Effectiveness of App-Based Cognitive Screening for Dementia by Lay Health Workers in Low Resource Settings. A Validation and Feasibility Study in Rural Tanzania [file App_revision_supplementary_tables.pdf]

Supplementary online tables

1. CERAD 10-word learning list delayed recall items and dementia

Total number of words recalled/10	DSM-V Dementia in stage 2 assessment (n = 105)	DSM-V No dementia in stage 2 assessment (n = 505)
0	71 (67.6%)	172 (34.05)
1	15 (14.3%)	59 (11.7%)
2	12 (11.4%)	86 (17.0%)
3	7 (2.86%	53 (10.5%)
4	0	40 (7.9%)
5	0	54 (10.7%)
6	0	20 (4.0%)
7	0	9 (1.78%)
8	0	5 (1.0%)
9	0	5 (1.0%)
10	0	2 (0.4%)

2. Animal naming performance and dementia

Number of animals named/minute	DSM-V Dementia in stage 2 assessment (n = 105)	DSM-V No dementia in stage 2 assessment (n = 505)
0	25 (35.2%)	46 (9.1%)
1	2 (33.3%)	4 (0.8%)
2	6 (35.3%)	11 (2.2%)
3	9 (25.7%)	26 (5.1%)
4	10 (25.0%)	30 (5.9%)
5	10 (18.5%)	44 (8.7%)
6	15 (25.0%)	45 (8.9%)
7	11 (25.0%)	33 (6.5%)
8	8 (16.0%)	42 (8.3%)
9	2 (4.3%)	45 (8.9%)
10	4 (6.0%)	63 (12.5%)
11	0	25 (5.0%)
12	1 (4.0%)	24 (4.8%)
13	1 (4.8%)	20 (4.0%)
14	0	12 (2.4%)
15	0	7 (1.4%)
16	0	15 (3.0%)
17	0	1 (0.2%)
18	1 (25.0%)	3 (0.6%)
≥ 19	0	9 (1.8%)
