# Supplementary material for: Effectiveness of App-Based Cognitive Screening for Dementia by Lay Health Workers in Low Resource Settings. A Validation and Feasibility Study in Rural Tanzania
Source: J Geriatr Psychiatry Neurol. 2020 Sep 23;34(6):613–21. doi: 10.1177/0891988720957105 (PMC8600584; doi:10.1177/0891988720957105)
Supplement: Supplemental Material, ODK_word_doc_screening_paper - Effectiveness of App-Based Cognitive Screening for Dementia by Lay Health Workers in Low Resource Settings. A Validation and Feasibility Study in Rural Tanzania [file ODK_word_doc_screening_paper.pdf]

type	name	label::English	label::Kiswahili	hint::English	hint::Kiswahili
start	start_time				
end	end_time				
note	instructions1	###You are at the start of the IDEA Cognitive Screening Assessment Battery. Swipe you finger across the screen to go forward to the next question or back to the previous question. You can go backward and change answers until you get to the final screen when you will be asked to finalize and save the completed form. **At this point no additional changes can be made**	###Uko katika mwanzo wa uchunguzi wa IDEA betrii. pangusa kwa kidole chako kwenye kioo cha simu yako ili kuendelea mbele kwenye swali lifuatalo au kurudi kwenye swali la nyuma. Unaweza kurudia kufanya masahihisho ya majibu na ukishamaliza kuisevu kabisa ** baada yahapo huwezi kufanyatena mabadiliko yoyote kwenye form **	This work is licensed under a Creative Commons Attribution-ShareAlike 4.0 International License (CC BY-SA 4.0)	This work is licensed under a Creative Commons Attribution-ShareAlike 4.0 International License (CC BY-SA 4.0)
begin group	section1General	Section 1. Background details	Sehemu ya 1. Maelezo ya wasifu		
note	note11	Record the following information before approaching the household	Andika vitu vifuatavyo kabla ya kuingia kwenye kaya.		
text	hhid	1.1. Please assign a unique participant ID	1.1. Andika namba ya mshiriki		
date	date	1.2. What is today's date?	1.2. Andika tarehe ya leo		
select_one interview_location	interview_location	1.3. Where is this interview taking place?	1.3. Ni mahali gani Mahojiano haya yanafanyika	Participants home/name of health centre or hospital	Nyumbanikwa mshiriki? /Jina la kituo cha afya au hospitali.

text	interview_location_other	1.3a. If you answered other to interview location, please state	1.3a. Kama jibu ni mwenye nyumba' kwenye swali lililopita, tafadhali fafanua	If you answered 'Other' to interview location, please state	Kama umejibu 'mengineyo' kwenye eneo la mahojiano, tafadhali elezea.
text	village	1.4. Please enter the name of the town or village where the person lives	1.4. Tafadhali ingiza jina la mji au kijiji ambapo mtu anaishi		
text	balozi	1.5. Please enter the name of the community/tribal leader	1.5. Tafadhali ingiza jina la jamii/ Balozi		
note	note12	Please record the following information once you have met the patient and introduced the study	Tafadhali andika maelezo yafuatayo mara unapokutana na mgonjwa na umekwisha muelezea kuhusu lengo la utafiti.		
select_one yes_no	consent	###1.6. Has the participant given written consent (signature or thumbprint) or has a close relative given assent for the participant to be included in the study	###1.6. Mshiriki ameshapewa karatasi ya ridhaa ya kushiriki (saini au dolegumba) au ndugu wa karibu amepewa karatasi ya kuridhia ndugu yake kushiriki katika utafiti.	##Assent should be taken if the participant lacks mental capacity to consent. **Do not proceed any further unless consent or assent is given**	##Ndugu wa karibu anaweza kusaini kama mshiriki anatatizo la akili hawezi kutoa ridhaa. **Usiendelee mbele mpaka ameridhia au ridhaa imetoewa kwa ndugu wa karibu imetolewa.**
geopoint	hhgps	1.7. Please record the GPS coordinates of this home.	1.7. Tafadhali andika GPS Coordinates za hii nyumba	If the GPS does not work after one minute of trying to record the location then this can be missed out.	Kama GPS kwa muda wa dakika moja haitakua inafanya kazi baada ya kujaribu kurekodi eneo ulipo hii inaweza kuachwa.
image	hhptpicture	1.8. If available, you may want to take a photo of the participant	1.8. Kama yupo, unaweza kutaka kuchukua picha ya mshiriki		

image	hhpicture	1.9. If available, you may want to take a photo of the front of the house	1.9. Kama yupo, unaweza kutaka kuchukua picha ya mbele ya nyumba.		
note	laminated_number	Please hand the patient their laminated study number	Tafadhali mkabidhi mshiriki kadi yenye number ya ushiriki kwenye utafiti		
end group					
begin group	section2	Section 2. Participant information	Sehemu ya 2. Maelezo ya mshiriki		
note	note1	Begin the interview with a short introduction about the test	Anza mahojiano kwa kufafanua kwa kifupi kuhusu hiki cha kumbukumbu	###I am going to ask you a few questions and test your memory and thinking. It will take around 20 minutes, but you can stop or have a rest at any point if you feel tired or unwell.	###Nitaenda kukuuliza maswali machache na kupima kumbukumbu yako na ufikiri. Itachukua kama dakika 20, lakini unaweza kuacha au kupumzika wakati wowote kama utajisikia kuchoka au kutojisikia vizuri
text	firstname	2.1. What is the participant's first name?	2.1. Jina la kwanza la mshiriki		
text	secondname	2.2. What is the participant's second or middle name (if they have one)?	2.2. Jina la pili la mshiriki	If they don't have a middle name, please leave blank	Kama hatakua na jina la katikati acha wazi.
text	lastname	2.3. What is the participant's last or third name?	2.3 Jina la Ukoo la mshiriki		
select_one gender	gender	2.4. Is the participant male or female?	2.4. Mwanaume/mwanamke ?		

select_one education	education	2.5. What is the participant's highest level of education?	2.5. Kiwango cha juu cha elimu cha mshiriki		
integer	educationyears	2.6. For how many years in total has the participant attended school or higher education?	2.6. Ni miaka mingapi kwa ujumla mshiriki amehudhuria shuleni au elimu ya juu.	This is their total years in education, including any vocational training, apprenticeships or adult courses	Hii ni jumla ya miaka yao ya elimu, ikiwa ni pamoja na mafunzo ya ufundi, ya kujiendeleza au elimu ya watu wazima.
select_one literacy	literacy	2.7. Has the participant ever learnt to read and write?	2.7. Mshiriki anajua kusoma na kuandika?Has the participant ever learnt to read and write?	This means now or in the past, read and write a simple note. If the participant could do this previously but cannot now due to eyesight or another health problem, answer yes.	Hii inamaanisha sasaau wakatiiuliopita, kusoma na kuandika neno rahisi.kama mshiriki aliweza kufanya hivyo hapo nyuma lakin hawezi kwa sasa kwasababu ya macho haoni au matatizo mengine, jibu Ndio.
select_one yes_no	AgeKnown	2.8. Does the participant know their age or year of birth?	2.8. Mshiriki anajua miaka yake au mwaka aliozaliwa?	If the participant does not know, but the relative does know, answer yes.	Kama mshiriki hajui, lakini ndugu wa karibu anajua, jibu ndio.
integer	birth_year	2.8a. In which year was the participant born?	2.8a Andika mwaka wa kuzaliwa wa mshirikiRekodi mwaka wa kuzaliwa wa mshiriki	Record as a four digit number (e.g. 1946)	Andika kama namba nne kama (mfano. 1946)
integer	participantage	2.8b. How old is the participant in years?	2.8b. Andika umri wa mshiriki kwa miakaRekodi umri wa mshiriki		
integer	estimate_age	2.8a. What is the estimated age of the participant based on use of memory prompts?	2.8a. Mshiriki anawastani wa umri gani kwa kutumia kipimo cha kumbukumbu.	Use prompts such as, 'Can you remember independence?' or other significant local or national events that	Tumia kianzio kama, ' unakumbuka uhuru'? Au lingine kama kitu kinachojulikana kiasilia au tukio la kitaifa ambalo kila mtu analikumbuka.

				everyone would remember	
select_one continue_working	workorretirement	2.9. Is the participant still working?	2.9. Mshiriki bado ni mfanyakazi?		
select_multiple occupation	occupation	2.10. What does the participant do for a living or what did they do for a living if retired?	2.10. Mshiriki anafanya kazi gani ya kumuwezesha kuishi au kama atastaafu anategemea kufanya nini?	Please tick all that apply	Tafadhali weka vema panapohusika
text	occupation_other	2.10a. If you answered 'Other' to the previous question about work, please state	2.10a. Kama jibu ni 'mengineyo' kwenye swali lililopita kuhusu kazi, tafadhali eleza.		
end group					
begin group	section3	Section 3. Contact information	Sehemu ya 3. Maelezo wakati tumekutana		
note	note3	Here is my contact information if you want to reach me	Kama utapenda kuwasiliana nami hii ni anuani yangu.	Give information sheet and contact details	Mpe fomu ya maelezo na njia ya mawasiliano
select_one yes_no	IDS_informant_present	3.1. Is an informant present?	3.1. kuna anaetoa maelezo kwa niaba ya mshiriki yuko hapa.	If an informant is available by phone that is ok. The accuracy of the interview will be improved by having an informant present.	Kama ndugu wa mshiriki anapatikana kwa simu ni sawa. Usahihi wa mahojiano utaboreshwa kwa kuwa na ndugu wa mshiriki
select_one informant	informant	3.2. What is the informant's relationship to the participant?	3.2. Mtoa maelezo anauhusiano gani na mshiriki?		
text	informant_other	3.2a. If you answered 'Other' to the previous question about the informants relationship, please state	3.2a. Kama amejibu 'mengineyo' kwenye swali lililopita kuhusu uhusiano wake na mshiriki, tafadhali elezea.		

select_one yes_no	contact	3.3. Ask the participant, **Please could we have a contact telephone number for you or a relative friend or neighbour?**	3.3. Uliza mshiriki, **Tafadhali tunaweza kupata namba ya simu yako au ya ndugu/rafiki wa karibu au jirani?**		
text	phone	3.4. Enter the participant's phone number or other contact	3.4. Ingiza number ya simu ya mshiriki au mawasiliano mengine		
end group					
begin group	section4	Section 4. IDEA cognitive screen	Sehemu ya 4. Kipimo cha IDEA uchunguzi wa cognitive		
note		Please read out the following text to the participant: I would like to ask you some questions about your memory and thinking. Please do not worry if you cannot answer a question. Some questions are more difficult than others.	Tafadhali soma maelezo yafuatayo kwa mshiriki: Ningependa kukuuliza maswali kidogo kuhusiana na kumbukumbu na uwezo wako wa kufikiria. Tafadhali usiwe na wasiwasi kama hutaweza kujibu swali. Kuna maswali mengine magumu kuliko mengine.		
select_multiple idea_screen_wordlist	IDSWORDREAD1	4.1. Please read out the following text to the participant: **I am going to read out a list of words. Please listen carefully and I will ask you to repeat them back to me once I have finished (read out the	4.1. Tafadhali soma maneno kwa mshiriki: **Nitakusomea orodha ya vitu kumi. Naomba uzisikilize kwa makini nanitakwambie uyarudie tena baada yangu pale nitakapo maliza (soma maneno hapo chini	Read out the words on the list slowly in the local language and tick the ones the participant can remember	soma maneno kwenye orodha taratibu kwa lugha ya asili,na na utie vema zile ambazo mshiriki amekumbuka.

		words below slowly). Please repeat back as many words as you can remember. We are going to do this three times. I will read it now for the first time**	taratibu). Tafadhali rudia tena maneno mara nyingine kwa kadri uwezavyo kukumbuka. Tutafanya hivi mara tatu.**		
select_one idea_screen_initialrecall	IDS_wordlist_initialrecall_1	4.2. How many of the words did the participant remember on the first attempt?	4.2. [JINA] NI maneno mangapi mshiriki amekumbuka kwenye jaribio la kwanza?		
select_multiple idea_screen_wordlist	IDSWORDREAD2	4.3. Please read out the following text to the participant: **Now I will read out the words again, listen carefully and I will ask you to repeat as many as you can. Now tell me all the words you can remember.**	4.3. Tafadhali soma tena maneno hayo kwa mshiriki: Sasa hivi nitakusomea ile orodha ya maneno tena, naomba uzisikilize kwa makini na niatkuhitaji urudie maneno mengi kadri uwezavyo. Sasa niambie maneno yote unayoyakumbuka.	Read out the words on the list slowly in the local language and tick the ones the participant can remember	soma maneno kwenye orodha taratibu kwa lugha ya asili,na na utie vema zile ambazo mshiriki amekumbuka.
select_one idea_screen_initialrecall	IDS_wordlist_initialrecall_2	4.4. How many of the words did the participant remember on the second attempt?	4.4 [JINA] Mshiriki amekumbuka maneno mangapi kwenye jaribio la pili?		
select_multiple idea_screen_wordlist	IDSWORDREAD3	4.5. Please read out the following text to the participant: **Now I will read out the words again one last time, listen carefully and I will ask you to repeat as many as you can. Now tell me all	4.5. Tafadhali soma maneno yafuatayo kwa mshiriki: Sasa nitasoma tena orodha ya maneno kwa mara ya mwisho, sikiliza kwa makini na nitakuambia urudie maneno yote kadri unavyoweza. sasa nitajie	Read out the words on the list slowly in the local language and tick the ones the participant can remember	soma maneno kwenye orodha taratibu kwa lugha ya asili,na na utie vema zile ambazo mshiriki amekumbuka.

		the words you can remember.**	maneno yote unayoweza kukumbuka.		
select_one idea_screen_initialrecall	IDS_wordlist_initialrecall_3	4.6. How many of the words did the participant remember on the third attempt?	4.6. [JINA] alikumbuka maneno mangapi kwenye jaribio la tatu?		
select_one idea_screen_correct2_incorrect	IDS_Q1bridge	4.7. Please read out the following text to the participant: **I will tell you the name of something and I want you to describe what it is. What is a bridge?**	4.7. Nitakuambia jina la kitu fulani na ninataka unielezee maana ya hicho kitu. Daraja ni nini?	Correct answer: Something that goes over a river,canyon or road. If participant is unsure - say 'imagine I am a small child and I have never seen a bridge. How would you explain it to me?'	Swali sahihi:kitu ambacho kinatembea juu ya maji, angani au barabarani. kama mshiriki hana uhakika-mwambie fikiria mimi ni mtoto mdogo na sijawahi kuona daraja. utaelezeaje hiyo mimi.
note	note4	####Remember to get a stopwatch ready before you start the next item. You may also need a pen and piece of paper to record the number of animals.	####Kumbuka kuandaa saa yako tayari kabla ya kuanza kipengele kinachofuata. Unaweza pia kuhitaji kalamu na karatasi kuandika namba ya wanyama.		
integer	IDS_Q2animals_number	4.8. Please read out the following text to the participant: **Please name as many wild or domestic animals as you can in one minute, and record the number of animals named below.**	4.8. Tafadhali soma maelezo yafuatayo kwa mshiriki.Tafadhali nitajie majina ya wanyama wa porini au wale wanaofugwa nuymbani, nitajie wengi kadri uwezavyo kwa dakika moja, na uandike namba ya wanyama waliotajwahapo chini	If participant stops before one minute, encourage to continue.	Kama mshiriki ataacha kabla ya dakika moja, mpe moyo aweze kuendelea.

select_one idea_screen_animals	IDS_Q2animals	4.9. Record the number of animals named again below	4.9. Andika number ya wanyama aliyoweza kutaja.	This is for the purposes of scoring	Hii ni kwaajili ya kupatia alama
select_one idea_screen_correct1_incorrect	IDS_Q3leader	4.10. Ask the participant: **Who is the chairman of your village? (or street leader)**	4.10. Muulize mshiriki, akutajie jina la Mwenyekiti wa kijiji chake anaitwa nani?	Partial name (first name or last name) is allowed if correct	Jina la utani(jina la kwanza au lapili) linaruhusiwa kama ni sahihi.
select_one idea_screen_correct2_incorrect	IDS_Q4weekday	4.11. Ask the participant: **What day of the week is it?**	4.11. Muulize mshiriki, Leo ni siku gani?	Correct day must be given	Siku sahihi katika juma lazima aitaje.
select_one idea_screen_delayedrecall	IDS_Q5delayedrecall	4.12. Ask the participant: **How many words can you remember from the list we learned a few minutes ago?**	4.12. Ni maneno mangapi kati ya yale kumi, unayoweza kuyakumbuka kutoka kwenye orodha niliyoukusomea dakika chache zilizopita	If participant says I don't know, encourage them to try - remember just one.	Kama mshiriki atasema sijui, mshawishi aweze kujaribu- akumbuke hata moja
select_one idea_screen_initialrecall	IDS_Q5delayedrecall_number	4.13. Please record again the number of words recalled in the previous question	4.13. Tafadhali andika tena namba ya maneno aliyokumbuka katika swali lililopita	This is for the purposes of scoring	Hii ni kwaajili ya kutoa alama.
note	note5matchstickinstruction	Please read out the following text to the participant: **Please make this shape using the matchsticks provided. I will show you once and then you have to make the shape exactly.**	Tafadhali soma maneno yafuatayo kwa mshiriki: **Tafadhali tengeneza maumbo haya kwa kutumia njiti za kiberiti nilizokupatia. Nitakuonesha mara moja na utatakiwa kutengeneza kama nilivyotengeneza hilo umbo.**	###Make the shape using the matchsticks. Ask the participant to notice all the matchsticks are pointing upwards. Collect up all matchsticks and hand to participant. Do not show the participant a picture of the matchsticks. If participant has severe visual impairment you may use pencils with coloured ends.	###Tengeneza maumbo kwa kutumia njiti za kiberiti. muulize mshiriki kutambua njiti za kiberitizote zinazo angalia juu. kusanya njiti zote na mkabidhi mshiriki. Usimuoneshe mshiriki picha ya njiti za kiberiti. kama mshiriki ana tatizo kubwa la kutokuona unaweza kutumia penseli yenye rangi mwishoni.

select_one idea_screen_matchsticks_correct1_incorrect	IDS_Q6matchsticks_element1	4.14. Are the middle two matchsticks pointing the same way?	4.14. Vichwa vya njiti mbili vimelekea upande mmoja?		
select_one idea_screen_matchsticks_correct1_incorrect	IDS_Q6matchsticks_element2	4.15. Are the outside two matchsticks pointing at an angle?	4.15. Kwa upandewa nje njiti mbili zimeelekea kwenye kona?		
select_one idea_screen_matchsticks_correct1_incorrect	IDS_Q6matchsticks_element3	4.16. Are the matchstick heads orientated correctly?	4.16. Vichwa vya njiti vimeelekezwa sawa?		
image	matchstickpicture	4.17. Please take a photograph of the matchstick task attempt	4.17. Tafadhali piga picha ya njiti katika umbo lililotengenezwa.		
calculate	IDEAscreentotal				
note	display	Total for the IDEA screen is: ${IDEAscreentotal}	Alama ya jumla ya utafiti wa IDEA ni: ${IDEAscreentotal}		
select_multiple problems_IDEA	problems_IDEA	4.18. This is a question for you the interviewer: Did any of the following problems affect the ability of the participant to complete any of the assessments?	4.18. Hili ni swali lako muhojaji: Kuna kitu chochote kati ya vifuatavyo vimeharibu uwezekano wa mshiriki kumaliza uchunguzi wowote?	Please tick all that apply	Tafadhali weka vema kwa kinacho husika
text	problems_IDEA_other	4.18a. If you answered 'Other' to the previous question about probels with the IDEA screen, please state.	4.18a. Kama majibu ni tofauti 'mengineyo' kwenye swali la nyuma kuhusu kudadisi wa kipimo cha IDEA tafadhali elezea.		
text	notes	4.19. This is a question for you, the interviewer: Are there any other comment you wish to make about the interview?	4.19. Hili ni swali kwaajili yako, Muhojaji: Kuna maelezo mengine ungependa kutoa kuhusiana na mahojiano/ dodoso?		

end group					
begin group	section5	Section 5. IDEA IADL screen	Sehemu ya 5. Uchunguzi wa IDEA IADL		
note	IADL_instructions	Start of IADL questionnaire: For each of the following questions please ask "can the older person do this with no difficulty?"	Tafadhali muulize ndugu/mlezi wa mtu mzee kila swali. Kwa kila swali tafadhali uliza“ Je mgonjwa anaweza kufanya hivi bila shida?”		
select_one IADL_answers	IADL_works_home	5.1. Ask the informant: **Can they assist in small works of the house with no difficulty?**	5.1. Muulize ndugu wa mshiriki: **anasaidia kazi ndogo ndogo za nyumbani bila ugumu?**		
select_one IADL_answers	IADL_give_advice	5.2. Ask the informant: **Can they give advice?**	5.2. Muulize ndugu wa mshiriki: **huwa anatoa ushauri?**		
select_one IADL_answers	IADL_preside_feasts_ceremonies	5.3. Ask the informant: **Can they preside over feasts and ceremonies with no difficulty?**	5.3. Muulize ndugu wa mshiriki: **anasaidia katika maswala mazito kama sherehe bila ugumu?**		
calculate	IADLscreentotal				
note	display	Total for the IADL screen is: ${IADLscreentotal}	Jumla alama za uchunguzi wa IADL ni: ${IADLscreentotal}		
end group					
begin group	section6	Section 6. Subjective memory	Sehemu ya 6. Subjective memory		
note	smcinstructions	####The following questions are about the participant's feelings about their memory and	####Maswali yafuatayo yanahusu mshiriki na hisia kuhusu kumbukumbu na uwezo		

		ability to understand things. Allow the participant to answer the questions as far as possible, although the informant may contribute if the participant is unable to answer.	wake wa kuelewa vitu. mruhusu mshiriki kujibu maswali kadri inavyowezekana, ijapokua ndugu wa mshiriki anaweza kuchangia kama mshiriki hawezi kujibu.		
select_one yes_no	smc1	6.1. Ask the participant: **Has your memory become worse over the last year?**	6.1. Muulize mshiriki: **Kumbukumbu yako inaendelea kuwa mbaya kupita mwaka wa nyuma?**		
select_one yes_no	smc2	6.2. Ask the participant: **Do your memory problems cause you difficulties in your daily activities?**	6.2. Muulize mshiriki: **Matatizo yako ya kumbukumbu yamekusababishia ugumu katika kazi zako za kila siku?**		
end group					
begin group	section7	Section 7: Delirium	Sehemu ya saba 7: Uchunguzi wa Delirium		
select_one CAM4	CAM4	7.1. This is a question for you, the interviewer: How does the participant seem to you?	7.1. Hili ni swali kwaajili yako Muhojaji: Mshiriki anaonekanaje kwa mtazamo wako?		
select_one CAM1a	CAM1a	7.2. Ask the informant: **Was the person like this last week? **	7.2. Muulize ndugu wa karibu, **alikua hivi week iliyopita?**	This means confusion or change in mental ability, not just illness.	Hii inamaana ni mchanganyiko au nia mabadiliko katika uwezo wa kiakili, sio ugonjwa tu. Na uwezo wake wa kufikiri na mihemko iko kama kawaida kwa week

					kadhaa au kwa muda mrefu.
select_one CAM1b	CAM1b	7.3. Ask the informant: **Has there been a change or fluctuation in their presentation over the past few hours or days?**	7.3. Muulize ndugu wa karibu, **kumekua na mabadiliko ya hali ya kubadilika, badilika na kurudia hali ile ile kwa saa chache au siku chache zilizopita?**	Is the person sometimes OK and sometimes confused?	Wakatimwingine yuko mzima , yuko sawa na wakati mwingine anachanganyikiwa?
select_one CAM1b	CAM1c	7.3. Ask the informant: **Has there been a change or fluctuation in their presentation over the past few hours or days?**	7.3. Muulize ndugu wa karibu, **kumekua na mabadiliko ya hali au kubadilika na kurudia hali ile ile kwa sasa kwa masaa machache **	Is the person sometimes OK and sometimes confused?	Wakatimwingine yuko mzima , yuko sawa na wakati mwingine anachanganyikiwa?
calculate	Del1				
calculate	Del2				
calculate	Del3				
calculate	Del4				
calculate	Del5				
calculate	Del6				
calculate	Del8				
calculate	Del10				
note	displayDel1	Total for the delirium screen is: ${Del1}	Jumla ya alama za uchunguzi wa delirium: ${Del1}		
note	displayDel2	Total for the delirium screen is: ${Del2}	Jumla ya alama za uchunguzi wa delirium: ${Del2}		
note	displayDel3	Total for the delirium screen is: ${Del3}	Jumla ya alama za uchunguzi wa delirium: ${Del3}		

note	displayDel4	Total for the delirium screen is: ${Del4}	Jumla ya alama za uchunguzi wa delirium: ${Del4}		
note	displayDel5	Total for the delirium screen is: ${Del5}	Jumla ya alama za uchunguzi wa delirium: ${Del5}		
note	displayDel6	Total for the delirium screen is: ${Del6}	Jumla ya alama za uchunguzi wa delirium: ${Del6}		
note	displayDel8	Total for the delirium screen is: ${Del8}	Jumla ya alama za uchunguzi wa delirium: ${Del8}		
note	displayDel10	Total for the delirium screen is: ${Del10}	Jumla ya alama za uchunguzi wa delirium: ${Del10}		
end group					
begin group	section8	Section 8: Referral	Sehemu ya 8: Rufaa		
select_one yes_no	informant_reliable	This is a question for you, the interviewer: In your opinion did the informant give reliable answers to the questions about the participant?	Hili ni swali kwaajili yako, Muhojaji: Kwa maoni yako maelezo yametoa majibu yanayotegemewa kwamaswali kuhusu mshiriki?		
note	Delirium_present	###Delirium is possible, the participant should seek urgent medical attention to find out the cause	###Kuna uwezekano wa delirium, mshiriki anatakiwa kupatiwa huduma za kitabibu za haraka kutafuta chanzo cha tatizo		
note	Delirium_not_present	###Participant does not appear to have delirium	###Mshiriki haoneshi kuwa na tatizo la delirium, hakuna hatua	###No further action is needed	###Hakuna chochote zaidi kinachotakiwa.

			yeyote inayotakiwa kuchukuliwa		
select_one yes_no	Medical_advice	Does the participant agree to seek medical attention?	Mshiriki amekubali kutafuta huduma za haraka za kitabibu kuhusiana na delirium?		
calculate	overall_cog_screen_total				
note	Total_cog_1	Total for the complete cognitive screen is: ${overall_cog_screen_total}	Jumla ya alama baada ya kumaliza uchunguzi wa cognitive ni: ${overall_cog_screen_total}		
note	Total_cog_2	Total for the complete cognitive screen is: ${IDEAscreentotal}	Jumla ya alama baada ya kumaliza uchunguzi wa cognitive ni: ${IDEAscreentotal}		
note	Screen_postive_dementia_1	###The participant has screened positive for possible dementia and should be assessed further	###Mshiriki katika uchunguzi amegundulika kuwa anatatizo la dementia na anatakiwa uchunguzi zaidi		
note	Screen_negative_dementia_1	###The participant has screened negative for possible dementia and does not require to be assessed further	###Mshiriki ambae kipimo kimeonyesha kuwa hana dementia na hatatahitaji uchunguzi zaidi		
note	Screen_postive_dementia_2	###The participant has screened positive for possible dementia and should be assessed further	###Mshiriki katika uchunguzi amegundulika kuwa anatatizo la dementia na hatakiwi uchunguzi zaidi		
note	Screen_negative_dementia_2	###The participant has screened negative for possible dementia and	###Mshiriki ameonekana kuwa hana tatizo la dementia na hahitaji uchunguzi zaidi.		

		does not require to be assessed further			
end group					
note	note81	#Send text message to participant thanking them for taking part	#Tuma message kwa mshiriki na umshukuru kwa kushiriki.		
note	note82	#Thank the person for their time	#Mshukuru mshiriki kwa muda wake.		
